# Antioxidant properties of lutein contribute to the protection against lipopolysaccharide-induced uveitis in mice

**DOI:** 10.1186/1749-8546-6-38

**Published:** 2011-10-31

**Authors:** Rong-Rong He, Bun Tsoi, Fang Lan, Nan Yao, Xin-Sheng Yao, Hiroshi Kurihara

**Affiliations:** 1 Institute of Traditional Chinese Medicine and Natural Products, Jinan University, Guangzhou 510632, China

## Abstract

**Background:**

Lutein is an important eye-protective nutrient. This study investigates the protective effects and mechanisms of lutein on lipopolysaccharides (LPS)-induced uveitis in mice.

**Methods:**

Lutein, suspended in drinking water at a final concentration of 12.5 and 25 mg/mL, was administered to mice at 0.1 mL/10 g body weight for five consecutive days. Control and model group received drinking water only. Uveitis was induced by injecting LPS (100 mg per mouse) into the footpad in the model and lutein groups on day 5 after the last drug administration. Eyes of the mice were collected 24 hours after the LPS injection for the detection of indicators using commercial kits and reverse transcription-polymerase chain reaction.

**Results:**

LPS-induced uveitis was confirmed by significant pathological damage and increased the nitric oxide level in eye tissue of *BALB/C *mice 24 hours after the footpad injection. The elevated nitric oxide level was significantly reduced by oral administration of lutein (125 and 500 mg/kg/d for five days) before LPS injection. Moreover, lutein decreased the malondialdehyde content, increased the oxygen radical absorbance capacity level, glutathione, the vitamin C contents and total superoxide dismutase (SOD) and glutathione peroxidase (GPx) activities. Lutein further increased expressions of copper-zinc SOD, manganese SOD and GPx mRNA. **Conclusion **The antioxidant properties of lutein contribute to the protection against LPS-induced uveitis, partially through the intervention of inflammation process.

## Background

Uveitis, a common ophthalmic disorder responsible for approximately 10% of blindness in western countries [1,2], may be caused by autoimmune disorders, infections or exposure to toxins. In experimental research, injection of endotoxins such as lipopolysacharide (LPS) is used to induce uveitis in susceptible animal species such as rats and mice [3,4]. LPS-induced uveitis in rodents mimics human uveitis, and is used as an animal model of pathogenesis of uveitis to evaluate the therapeutic efficacy of drugs [5]. Increased inflammatory markers such as cyclooxygenase-2 (COX-2) and inducible-nitric oxide synthase (iNOS) result in the breakdown of blood-ocular barrier and infiltration of leukocytes into ocular tissues, leading to LPS-induced uveitis [6]. Inflammation is considered as the cause for the LPS-induced uveitis. Previous research indicated that LPS initiated a cascade of signaling reactions not only to the expression of inflammatory cytokines, chemokines and other inflammatory markers, but also activated redox-sensitive transcription factors and increased the reactive oxygen species (ROS) level [7]. However, little attention has been paid to the role of oxidative stress in the development of LPS-induced uveitis.

Recently, we confirmed that ROS plays an important role in mediating the inflammatory signals induced by LPS and that natural antioxidants exert protective effects on the LPS-induced uveitis [8]. Meanwhile, we screened various natural products using the LPS-induced mouse uveitis model and found that lutein possesses the most potent bioactivities.

Lutein, concentrated in a small area of the retina called macula, protects the eye against oxidative stress and is one of the most commonly found carotenoids in fruits and vegetables [9]. In recent years, research has been focused on the importance of lutein in eye health, such as protecting vision and cataracts [10]. Studies found that an increase in macula pigmentation lowers the risk for eye diseases and that lutein intake is correlated with pigmentation in eyes [11,12]. Randomized clinical trials found that visual functions are improved by lutein alone or lutein-included nutrients [13]. Inhibitory effects of lutein on the LPS-induced uveitis in rats were also reported and mechanistic studies on the mouse macrophage cell line (RAW264.7 cells) were carried out [9].

The antioxidant properties of lutein may have protective effects against LPS-induced uveitis. To confirm this, the present study aims to investigate the effects of lutein on the changes of lipid peroxidation products, antioxidant capacity and gene expressions of antioxidase in the whole eye of mice.

## Methods

### Chemicals

Lutein was supplied by JF-Natural Ltd. (China) (batch No: 0903110-24). Commercial kits of Coomassie brilliant blue, malondialdehyde (MDA), total superoxide dismutase (SOD) and glutathione peroxidase (GPx) were purchased from Jiancheng Bioengineering Institute (China). LPS from *Salmonella typhimurium *was purchased from Sigma-Aldrich (USA). Glutathione (GSH) was purchased from Kohjin Co Ltd (Japan).

### Animals and treatments

Seven-week-old male *BALB/C *mice were purchased from Guangdong Medical Laboratory Animal Center (China). Mice were kept in a specific room with room temperature at 23 ± 1°C and a 12-hour light-dark cycle (lights on from 06:00 to 18:00). Prior to the experiments, the animals were acclimatized for one week with standard laboratory diet and water. Mice were then arbitrarily divided into four groups of 18 animals each. Experimental groups received oral administration of lutein suspended in drinking water at a concentration of 12.5 and 25 mg/mL, while control and model group received drinking water only. Intake of the lutein suspension was 0.1 mL/10 g body weight for five consecutive days. On the fifth day, uveitis was induced in the experimental groups by injecting LPS diluted in saline at 100 mg per mouse into the mouse footpad. All mice were sacrificed 24 hours after the injection of LPS in an ether atmosphere and the eyes were collected immediately. The care and treatment of the animals were conducted in accordance with the Guide for the Care and Use of Laboratory Animals by the United States National Institutes of Health (NIH publication no. 85-23, revised 1985).

### Measurement of the nitric oxide level

Nitric oxide (NO) level of 40% eye homogenate was determined by the Griess method [14]. Briefly, a 40 μL sample was transferred into 96-well microplates, and 160 μL Griess reagent, consisting of 1% sulfanilamide, 0.1% N-(1-naphthyl) ethylenediamine hydrochloride and 2.5% H_3_PO_4_, was added at room temperature. After 20 minutes, the purple azo-dye was detected at 540 nm with a MK3 microplate reader (Labsystems, Finland).

### Measurement of the MDA and ORAC levels

We used an MDA commercial kit (Jiancheng Bioengineering Institute, China) to measure the MDA level of the 40% eye homogenate. We measured the ORAC level of the 4% eye homogenate using a previously described method [15].

### Measurement of the GSH and vitamin C levels

The 40% eye homogenate was deproteinized by adding 3% PCA (1:1) and then centrifuged at 12,000 rpm (13201×*g*, Sigma, USA) for 15 minutes at 4°C. The supernatant was filtered through a 0.45 μm filter disk. Glutathione and vitamin C levels in the filtrate were determined by high-performance liquid chromatography (HPLC, Hitachi, Japan) as previously described [8].

### Measurement of SOD and GPx activities

Protein concentration of eye homogenate (1%) was determined with a Coomassie brilliant blue kit (Jiancheng Bioengineering Institute, China). The total superoxide dismutase and glutathione activities were detected using 40% eye homogenate by SOD and GPx kits (Jiancheng Bioengineering Institute, China).

### Measurement of mRNA expression of SOD and GPx

Gene expression levels of antioxidase were semi-quantitatively assessed with reverse transcription-polymerase chain reaction (RT-PCR). Total RNA was extracted with the Trizol reagent from the samples according to the manufacturer's protocol (Invitrogen, USA). A total of 3 μg of RNA was reverse-transcribed into cDNA at 42°C for one hour in 20 μL reaction mixture containing mouse moloney leukemia virus reverse transcriptase (Tiangen, China) with oligo (dT)15 primer (Tiangen, China), followed by polymerase chain reaction (PCR). PCR (Veriti, USA) was performed with 1 μL cDNA, 2.5 μL 10 × Taq reaction buffer (Tiangen, China), 2 μL dNTP mixture, 1 μM forward primer, 1 μM reverse primer, 1 μL Taq polymerase (Tiangen, China) in a total volume of 25 μL. The cDNA was amplified with specific primers for 30 cycles, beginning at 94°C for 30 seconds, then at an annealing temperature of 58°C for 40 seconds, and 72°C for 50 seconds, with final incubation at 72°C for seven minutes. The sequences of PCR primers are shown in Table 1. The PCR products were electrophoresed on a 1% agarose gel and visualized with ethidium bromide staining. The band intensity of ethidium bromide fluorescence was measured with an image analysis system (Bio-Rad, USA), and then quantified with Quantity One analysis software (Bio-Rad, USA) and expressed as the ratio to *β*-actin.

**Table 1:** Sequences of the oligonucleotides used as primers

mRNA	Sequence (5' to 3')	Length (base pair)	**Accession no**.
CuZnSOD	F: 5'-ATGGCGATGAAAGCGGTGTG-3' R: 5'-TTACTGCGCAATCCCAATCAC-3'	456	NM_011434
MnSOD	F: 5'-AAGCACAGCCTCCCAGACCT-3' R: 5'-TCACTTCTTGCAAGCTGTGTATCTT-3'	597	NM_013671
GPx	F: 5'-GAAGTGCGAAGTGAATGG-3' R: 5'-TGGGACAGCAGGGTTT-3'	255	NM_008160
β-actin	F: 5'-GAGGGAAATCGTGCGTGAC-3' R: 5'-GCTGGAAGGTGGACAGTGAG-3'	446	NM_007393

GenBank accession numbers of cDNA and corresponding genes are available at http://www.ncbi.nlm.nih.gov/.

F: forward primer; R: reverse primer

### Statistical analysis

The data were presented as mean ± standard deviation (SD). Statistical analysis of data was performed with an SPSS 13.0 statistical package (IBM, USA). One-way analysis of variance (ANOVA) was used to analyze data differences among groups, followed by Dunnett's significant *post-hoc *test for correcting multiple pair-wise comparisons. Differences were considered as statistically significant when *P *< 0.05.

## Results

### Effects of lutein on the NO levels

**Figure 1:**
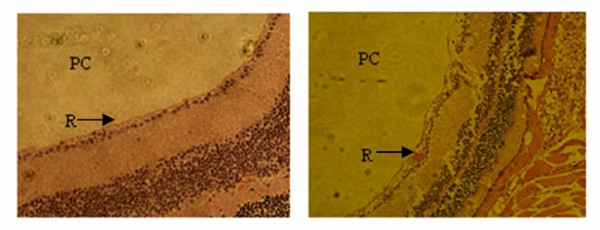
**Microscopic image of the eye tissues obtained from the LPS-treated mice**. Photomicrographs of hematoxylin-eosin-stained sections of eyes from (A) control group and (B) model group 24 hours after the LPS injection. Views of the posterior chamber (PC) and retina (R); magnification ×400

**Figure 2:**
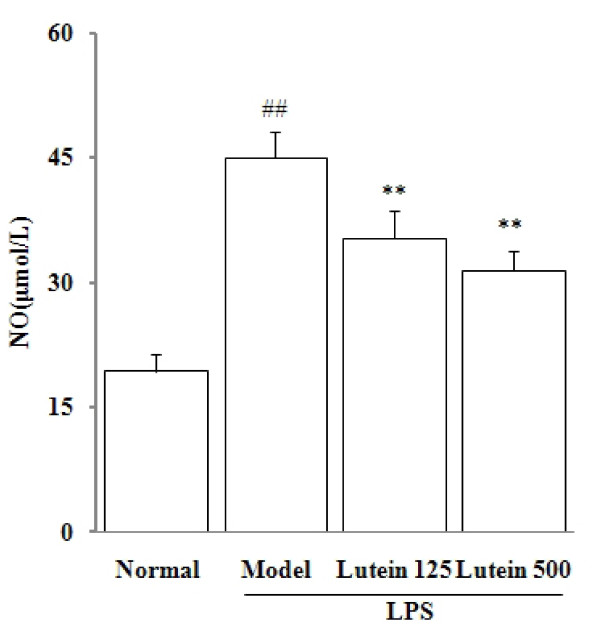
**Effects of lutein on the NO levels in the eyes of the mice treated with LPS**. Seven-week-old male *BALB/C *mice were injected with LPS in the footpad at 100 mg per mouse. The results are presented as mean ± SD obtained from 18 animals in each group. ##*P *< 0.01: significantly different from the control group and **P < 0.01: from the model group

The LPS-induced uveitis was confirmed by significant retinal edema and hemorrhage (Figure 1). The anti-inflammatory effects of lutein on the LPS-induced uveitis were investigated by determining the NO levels in the eyes of the mice treated with LPS. As shown in Figure 2, the NO level in the eye tissue of the control mice was 19.3 ± 2.1 μmol/mL. An increase (44.9 ± 3.2 μmol/mL, *P *= 0.00138) was observed in the model mice. However, when mice were pre-treated with lutein (125 and 500 mg/kg/d for five days), the NO levels were significantly decreased to 35.2 ± 3.3 and 31.3 ± 2.5 μmol/mL (*P *= 0.00816; 0.00593) respectively.

### Effects of lutein on the MDA and ORAC levels

**Figure 3:**
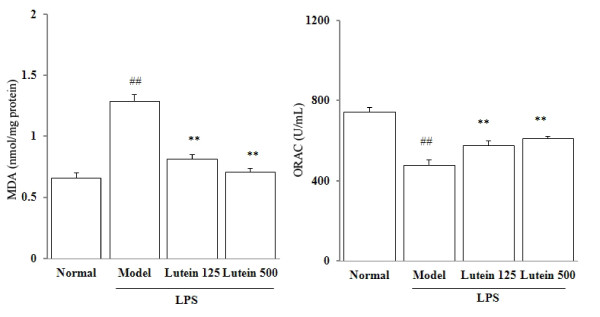
**Effects of lutein on the MDA and ORAC levels in the eyes of the mice treated with LPS**. Seven-week-old male *BALB/C *mice were injected with LPS in the footpad at 100 mg per mouse. The results are presented as mean ± SD obtained from 18 animals in each group. ##*P *< 0.01: significantly different from the control group and **P < 0.01: from the model group

MDA and ORAC levels of the eye homogenate are indicative of the oxidative damage and anti-oxidative capacity respectively. As shown in Figure 3A, the MDA levels in the eyes of the model group were significantly increased from 0.66 ± 0.04 to 1.29 ± 0.05 nmol per milligram of proteins (*P *= 0.00261). By contrast, the basal values of ORAC in the eye homogenate of the control mice were 744.5 ± 1.4 U/mL, and LPS injection decreased the ORAC level to 65% of the control group. However, lutein administrations (125 and 500 mg/kg/d for five days) to the LPS treated mice significantly reduced the MDA levels to 0.81 ± 0.04 and 0.71 ± 0.03 nmol/mg protein (*P *= 0.00468) (Figure 3A), and significantly raised the ORAC levels to 576.4 ± 21.6 and 608.0 ± 11.6 U/mL (*P *= 0.00597; 0.00384) respectively (Figure 3B).

### Effects of lutein on the GSH and vitamin C levels

**Figure 4:**
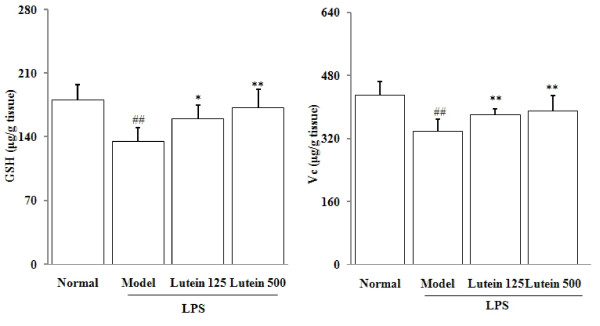
**Effects of lutein on the GSH and vitamin C levels in the eyes of the mice treated with LPS**. Seven-week-old male *BALB/C *mice were injected with LPS in the footpad at 100 mg per mouse. The results are presented as mean ± SD obtained from 18 animals in each group. ##*P *< 0.01: significantly different from the control group and *P < 0.05, **P < 0.01: from the model group

Small-molecule antioxidants (*ie *GSH and vitamin C) were detected in the eyes of the mice. As shown in Figure 4, the GSH levels in the eyes of the model group were significantly lowered from 180.3 ± 17.1 to 135.5 ± 15.2 μg/g tissues (*P *= 0.00732). Lutein administrations (125 and 500 mg/kg/d for five days) significantly increased the GSH level to 160.0 ± 15.3 and 172.0 ± 20.2 μg/g tissue (*P *= 0.02924; 0.00719) in the eyes of the mice treated with LPS. Similarly, LPS decreased the vitamin C levels from 430.2 ± 35 to 340.6 ± 29.0 μg per gram of tissue (*P *= 0.00618). Lutein administrations (125 and 500 mg/kg/d for five days) significantly lowered the vitamin C levels to 380.6 ± 15.0 and 390.7 ± 29.1 μg per gram of tissue (*P *= 0.00833; 0.00509) respectively.

### Effects of lutein on the total SOD and GPx activities

**Figure 5:**
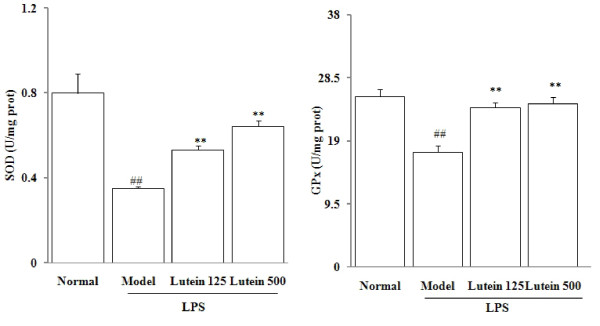
**Effects of lutein on the total SOD and GPx activities in the eyes of the mice treated with LPS**. Seven-week-old male *BALB/C *mice were injected with LPS in the footpad at 100 mg per mouse. The results are presented as mean ± SD obtained from 18 animals in each group. ##*P *< 0.01: significantly different from the control group and **P < 0.01: from the model group

The activities of free oxygen radical scavenger enzymes were investigated by determining the SOD and GPx activities in the eyes of the mice treated with LPS. Total SOD activity in the model group was significantly lowered to 0.35 ± 0.01 U per milligram of proteins (*P *= 0.00604). Similarly, LPS decreased the GPx activities to 17.3 ± 0.9 U per milligram of proteins (*P *= 0.00788). However, lutein administrations (125 and 500 mg/kg/d for five days) increased the SOD activities to 0.5 ± 0.02 and 0.6 ± 0.03 U per milligram of protein (*P *= 0.00573; 0.00422), and raised the GPx activities to 24.02 ± 0.82 and 24.6 ± 1.0 U per milligram of protein (*P *= 0.00392; 0.00310) respectively (Figure 5).

### Effects of lutein on the expression of the CuZnSOD, MnSOD and GPx mRNA levels

**Figure 6:**
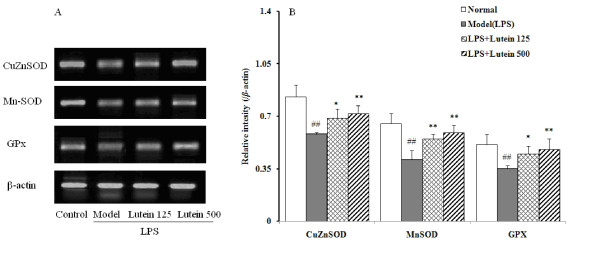
**Effects of lutein on the mRNA expression of CuZnSOD, MnSOD and GPx in the eyes of the mice treated with LPS**. (A) Agarose gel electrophoresis of RT-PCR amplication of CuZnSOD, MnSOD, GPx mRNA and β-actin mRNA. (B) Densiometric analysis of PCR products. Results were generated as relative intensity units by densitometry and expressed as the ratio to β-actin. The results are presented as mean ± SD obtained from 18 animals in each group. ##*P *< 0.01: significantly different from the control group and *P < 0.05, **P < 0.01: from the model group

Compared with the control group, all expression levels of CuZnSOD, MnSOD and GPx mRNA in the model group decreased (*P *= 0.00487; 0.00372; 0.00703). Lutein (125 and 500 mg/kg/d for five days) enhanced the mRNA expressions of CuZnSOD (*P *= 0.0237; 0.00858), MnSOD (*P *= 0.00879; 0.00635) and GPx (*P *= 0.0159; 0.00937) (Figure 6).

## Discussion

Our results indicate that cellular infiltration and protein leakage into the anterior chamber of mice eyes reached maximum at 24 hours after LPS injection at 100 mg/mouse. Cytokines play essential roles in the development of uveitis. LPS-induced uveitis is often accompanied by elevated expression of cytokines such as tumor necrosis factor-*α *(TNF-*α*), interleukin-6 (IL-6), monocyte chemoattractant protein-1 (MCP-1) [16]. Other inflammatory mediators such as NO and prostaglandin-E_2 _(PG-E_2_) are also involved in the pathogenesis of LPS-induced uveitis [17]. In this study, the LPS injection to the mice led to a massive release of NO in eye tissues. This increase of NO production may be due to the up-regulated iNOS mRNA expression in response to LPS [9]. NO is not only a pro-inflammatory mediator, but also a highly damaging ROS precursor [18]. Excess NO causes eye damage by the formation of cytotoxic peroxynitrite which modifies proteins by nitrating tyrosine residues, forming dityrosine and oxidizing tryptophan and cysteine [19]. Moreover, ROS production can be induced by LPS injection, while oxidative stress may promote the production of pro-inflammatory mediators thereby causing tissue damage [20].

We conducted experiments on the role of oxidative stress in the development of LPS-induced uveitis. We found a significant increase of MDA level and decrease of ORAC level in the eyes of the mice with LPS-induced uveitis. Endogenous antioxidants (*eg *GSH and vitamin C) help eye tissues increase their capability of ROS quenching [21]. Several studies reported that lutein intake elevates endogenous antioxidant expressions [22,23]. In the present study, LPS significantly depleted GSH and vitamin C and lowered the ORAC level by 35%, possibly leading to decreased antioxidant capacity. Our study demonstrated that oral administration of lutein significantly suppressed the MDA production and increased the diminished ORAC level in the eyes of the mice. Oral administration of lutein significantly recovered the GSH and vitamin C levels in the eyes of the LPS-treated mice, indicating that the antioxidant properties of lutein help protect against LPS-induced uveitis in mice. Furthermore, LPS decreased the total SOD and GPx activities, due to the down-regulation of the mRNA expressions of CuZnSOD, MnSOD and GPx. Lutein increased both the mRNA expressions and activities of SOD and GPx, suggesting that lutein can improve antioxidase activities at gene level in LPS-induced uveitis.

Lutein possesses higher antioxidant properties than other carotenoids. The antioxidant activities of lutein are almost 10- and 15-folds of those of *β*-carotene and lycopene [24]. The ED_50 _value of lutein as a free radical scavenger is 0.7 μM [25]. Rock *et al*. reported that the mean serum lutein concentration of human was about 0.2 μM under normal dietary intake [26]. As the retinal and macular concentration of lutein are raised by lutein-containing food [27], dietary supplement of lutein may elevate its concentration in tissue, thereby achieving its protective effects.

Apart from the ROS scavenger property of lutein [28,29], lutein suppresses the activation of the nuclear factor (NF)-κB and the degradation of the inhibitor-κB (IκB) [9]. NF-κB, one of the most ubiquitous transcription factors, has been suggested to play a key role in these reactions [30]. Both production and release of inflammatory cytokines and ROS induced by LPS depend on inducible gene expression mediated by the activation of NF-κB [31]. Under quiescent conditions, NF-κB is sequestered in the cytosol and bound to the IκB. However, once IκB is dissociated from the complex by lutein, NF-κB translocates into the nucleus, leading to decreased gene transcription of inflammatory cytokines, chemokines and other inflammatory markers such as iNOS, as well as redox-sensitive transcription factors [7]. While previous studies reported that lutein reduced the concentrations of NO, TNF-*α*, IL-6, PGE-2, MCP-1 and MIP-2 in aqueous humor [9], our results suggest that the eye-protective effects of lutein may be related to the alleviation of oxidative stress.

## Conclusion

The antioxidant properties of lutein contribute to the protection against LPS-induced uveitis, partially through the intervention of inflammation process.

## Abbreviations

LPS: lipopolyssacharide; NO: nitric oxide; ORAC: oxygen radical absorbance capacity; GSH: glutathione; SOD: superoxide dismutase; GPx: glutathione peroxidase; CuZnSOD: copper/zinc superoxide dismutase; MnSOD: manganese superoxide dismutase; COX-2: cyclooxygenase-2; iNOS: inducible-nitric oxide synthase; ROS: reactive oxygen species; HPLC: high-performance liquid chromatography; RT-PCR: reverse transcription-polymerase chain reaction; TNF-*α*: tumor necrosis factor-*α*; IL-6: interleukin-6; MCP-1: monocyte chemoattractant protein-1; PG-E2: prostaglandin-E2

## Competing interests

The authors declare that they have no competing interests.

## Authors' contributions

XSY and HK designed the study. RRH and BT performed the experiments and wrote the manuscript. FL and NY performed the experiments. HK also wrote the manuscript. All authors read and approved the final version of the manuscript.
